# Acceptability of Robotic-Assisted Exercise Coaching Among Diverse Youth: Pilot Study

**DOI:** 10.2196/12549

**Published:** 2019-07-31

**Authors:** Amelia K Barwise, Christi A Patten, Martha J Bock, Christine A Hughes, Tabetha A Brockman, Miguel A Valdez Soto, Chung-Il Wi, Young J Juhn, Daniel R Witt, Stephen Sinicrope, Samantha R Kreps, Henry D Saling, James A Levine, Joyce E Balls-Berry

**Affiliations:** 1 Clinical and Translational Science PhD Program Mayo Clinic Graduate School of Biomedical Sciences Mayo Clinic Rochester, MN United States; 2 Pulmonary and Critical Care Medicine Mayo Clinic Rochester, MN United States; 3 Department of Psychiatry and Psychology Behavioral Health Research Program Minnesota BioBusiness Center, Mayo Clinic Rochester, MN United States; 4 Center for Clinical and Translational Science,Community Engagement Program Department of Psychiatry and Psychology Minnesota BioBusiness Center, Mayo Clinic Rochester, MN United States; 5 Center for Clinical and Translational Science Community Engagement Program Mayo Clinic Rochester, MN United States; 6 Asthma Epidemiology Research Unit and Community Pediatric and Adolescent Medicine Mayo Clinic Rochester, MN United States; 7 Mayo Clinic Alix School of Medicine Mayo Clinic Rochester, MN United States; 8 Health Sciences University of Minnesota Rochester, MN United States; 9 Center for Clinical and Translational Science Community Engagement Program Minnesota BioBusiness Center, Mayo Clinic Rochester, MN United States; 10 Department of Laboratory Medicine and Pathology Mayo Clinic Rochester, MN United States; 11 Fondation Ipsen Paris France; 12 Division of Endocrinology Department of Medicine Case Western Reserve University Cleveland, OH United States; 13 Division of Epidemiology Department of Health Sciences Research Mayo Clinic College of Medicine and Science, Mayo Clinic Rochester, MN United States

**Keywords:** robotics, adolescents, exercise, coaching, physical activity, technology

## Abstract

**Background:**

Almost 80% of adolescents do not achieve 60 minutes or more of physical activity each day as recommended by current US national guidelines. There is a need to develop and promote interventions that increase physical activity among adolescents. With increased interest in digital technologies among adolescents, robotic-assisted platforms are a novel and engaging strategy to deliver physical activity interventions.

**Objective:**

This study sought to assess the potential acceptability of robotic-assisted exercise coaching among diverse youth and to explore demographic factors associated with acceptance.

**Methods:**

This pilot study used a cross-sectional survey design. We recruited adolescents aged 12-17 years at three community-based sites in Rochester, MN. Written informed consent was obtained from participants’ parents or guardians and participants gave consent. Participants watched a brief demonstration of the robotic system-human interface (ie, robotic human trainer). The exercise coaching was delivered in real time via an iPad tablet placed atop a mobile robotic wheel base and controlled remotely by the coach using an iOS device or computer. Following the demonstration, participants completed a 28-item survey that assessed sociodemographic information, smoking and depression history, weight, and exercise habits; the survey also included the eight-item Technology Acceptance Scale (TAS), a validated instrument used to assess perceived usefulness and ease of use of new technologies.

**Results:**

A total of 190 adolescents participated in this study. Of the participants, 54.5% were (103/189) male, 42.6% (81/190) were racial minorities, 5.8% (11/190) were Hispanic, and 28.4% (54/190) lived in a lower-income community. Their mean age was 15.0 years (SD 2.0). A total of 24.7% (47/190) of participants met national recommendations for physical activity. Their mean body mass index (BMI) was 21.8 kg/m^2^ (SD 4.0). Of note, 18.4% (35/190) experienced depression now or in the past. The mean TAS total score was 32.8 (SD 7.8) out of a possible score of 40, indicating high potential receptivity to the technology. No significant associations were detected between TAS score and gender, age, racial minority status, participant neighborhood, BMI, meeting national recommendations for physical activity levels, or depression history (*P*>.05 for all). Of interest, 67.8% (129/190) of participants agreed that they and their friends were likely to use the robot to help them exercise.

**Conclusions:**

This preliminary study found that among a racially and socioeconomically diverse group of adolescents, robotic-assisted exercise coaching is likely acceptable. The finding that all demographic groups represented had similarly high receptivity to the robotic human exercise trainer is encouraging for ultimate considerations of intervention scalability and reach among diverse adolescent populations. Next steps will be to evaluate consumer preferences for robotic-assisted exercise coaching (eg, location, duration, supervised or structured, choice of exercise, and/or lifestyle activity focus), develop the treatment protocol, and evaluate feasibility and consumer uptake of the intervention among diverse youth.

## Introduction

Engaging in regular physical activity is effective for reducing the risk of obesity and mitigating its negative impacts on health [1]. Routine physical activity is crucial for healthy growth and development and for establishing lifelong routines that promote health and well-being. Engaging in regular physical activity benefits cardiorespiratory fitness, promotes growth of strong bones, reduces anxiety and depressive symptoms, improves mental health, and help teens maintain a healthy weight [2,3].

The 2008 US Physical Activity Guidelines for Americans [4] recommends children and adolescents aged 6-17 years engage in at least one hour or more of physical activity daily, a goal that 79% of adolescents do not achieve [5]. Furthermore, as youth grow into adults, the proportion of those not meeting these guidelines increases [6-8]. Therefore, there is a need to develop methods and strategies to promote physical activity among adolescents [7].

Some work has been done to examine the role of technology to improve lifestyle habits among adolescents. A systematic review by Chen et al examined the efficacy of technology-based interventions for healthy weight management in adolescents, including interactive video gaming, tailored Web-based health information, and the use of Wii Fit (Nintendo) [9]. Overall, this review found increased physical activity and weight loss in the intervention groups. Lau et al’s systematic review of information and communication technology-based interventions for promoting physical activity behavior change in children and adolescents included studies examining the effect of the Internet, email, and short message service (SMS) text messaging as assistive modes to deliver interventions. This review found evidence to support the use of information and communication-based interventions for increasing physical activity among youth [10]. Limitations of included studies in these reviews were lack of long-term follow-up and limited measurement of intervention exposure (ie, engagement with interventions) [9,10]. A more recent systematic review found that SMS text messaging may increase physical activity, but specifics about effective intervention elements were inconclusive [11]. Among Hispanic and black youth, active video gaming was shown to be a potentially useful mechanism to increase physical activity [12]. Among adults, digital health coaching delivered through the Web or mobile phones (eg, texting or apps) is also effective for enhancing physical activity [13].

Robotic-assisted technologies are emerging, but their full potential to enhance lifestyle behavior has yet to be realized and some have expressed concerns about their limitations in specific scenarios [14,15]. Many of these technologies emulate, but do not include, the support and empathy of a live coach and this may be disquieting [16]. For example, a recent study utilizing a fully automated robot for motivational interviewing to increase physical activity found that while participants appreciated the novelty and nonjudgmental nature of the technology, their experience was limited by the lack of individualized responses from, or social interactions with, the robot [17].

Based on the literature, combining the components of digital technology with human interactions may be a useful approach. Delivering exercise interventions through a mobile robotic device is better than videoconferencing because it allows the coach to remotely move with and around the individual, providing instruction and correction of exercise form, reinforcement, and support. Robotic-assisted interventions, where the coach interacts in real time, could therefore bridge the gap between human and embodied support [18,19].

The objectives of this pilot study were to assess the potential acceptability of robotic exercise coaching among a sample of racially and socioeconomically diverse youth and explore demographic factors and other variables associated with acceptance.

## Methods

### Study Approval and Design

The study was approved by the Mayo Clinic Institutional Review Board. The study used a cross-sectional survey design.

### Recruitment and Participants

We displayed posters and ads in select community locations in Rochester, MN, and on websites and conducted face-to-face outreach between March 5 and June 11, 2018. We recruited a convenience sample of adolescents from three community settings that serve racially and socioeconomically diverse youth and offer programs after school and on weekends. Study team members consulted with the staff at each community-based organization to determine appropriate times for recruitment and data collection. Recruitment took place four times at the Rochester Public Library and three times at the YMCA, each lasting about three hours; a local church also hosted one recruitment event.

Study staff screened adolescents in person to determine their potential to participate based on the study eligibility criteria: (1) aged 12-17 years old and (2) possessing a completed written informed consent and assent document, signed by the parent or guardian and participant. Exclusion criteria were being older than 17 years old and younger than 12 years old. We did not track the number of adolescents who received consent forms but did not complete them.

### Materials

**Figure 1:**
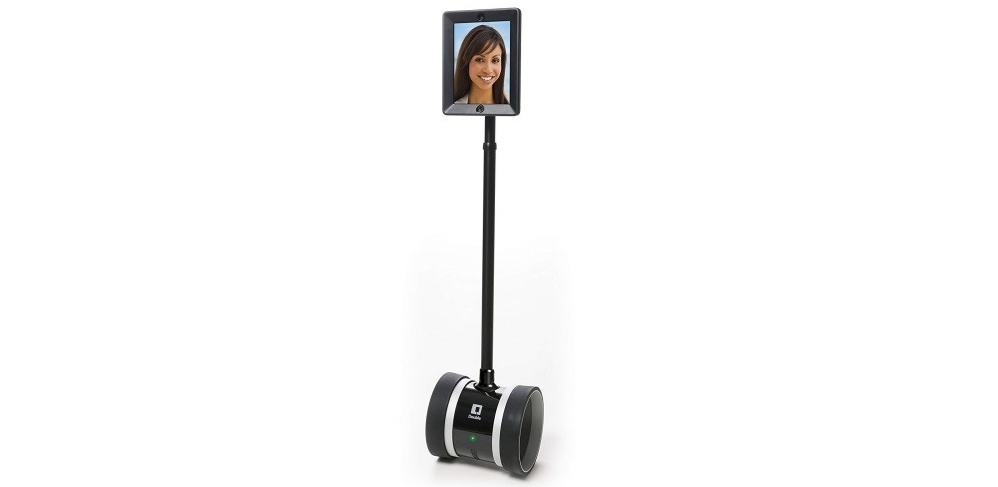
Robotic human-trainer technology.

The robotic system-human interface technology was used as a robotic-assisted exercise coach. The interface was delivered in real time via an iPad tablet placed on a mobile robotic wheel base and controlled remotely by an iOS device or computer. Developed by Double Robotics, Inc, for telecommuting and school attendance, the device measures 5 feet 1 inch in length. The “robot” iPad interface (see Figure 1) and the mobile phone device used to control it both required installation of the Double app (Double Robotics) [20,21].

Wi-Fi access on both devices was necessary to ensure functionality, which was available in each of the three community settings where the robot was demonstrated. A study staff member was designated as the exercise coach; she logged into the Double app through her mobile phone device and remotely interfaced with the iPad robot for demonstration purposes.

### Procedures

Prior to recruitment events, possible participants were identified at each setting by community and study staff, who gave a brief overview of the study to assess interest in participating. Those interested were asked to return the assent and consent forms, signed by the participant and his or her parent or guardian, on the day of recruitment. For some participants, these procedures all occurred on the same day. Once consent was obtained by study staff, participants observed the demonstration in a group setting in a private room with 7-10 other adolescents. The demonstration consisted of a 3-5-minute session during which a study team member played the role of the exercise coach.

The coach followed a script, included in Textbox 1 below, adapted from a prior study that assessed the acceptability of a robotic human trainer among diverse adults [22]. The script and anticipated intervention is based on social cognitive theory, including a focus on social support, reinforcement, and enhancing self-efficacy by providing feedback from the coach, along with emphasizing benefits of, and reducing barriers to, exercise [23]. The trainer began with a brief introduction of herself, a discussion about the benefits of exercise, and an explanation about the potential role of a robotic trainer, followed by a demonstration of the robotic trainer in motion and performing maneuvers. The script did not focus on a specific type of exercise or physical activity.

**Exercise coach script.**

Hi my name is _____ (coach). It’s nice to see you (all of you)!

As a robotic trainer, my role is to help people to exercise more, but I also focus my time on them and how they are doing. My role is to support and encourage people even while they are exercising. I try to bring positive energy to the exercise sessions and help them see all of the important progress they are making, even when it is tough going at times.

Let’s talk about some of the benefits of exercise for *you*. What is important to you?

That’s good! Anything else you have noticed? (if not already mentioned): Some people also say that exercise gives you:

- More energy
- Better sleep
- A sense of accomplishment or achievement
- A way to have fun
- A way to spend time with my friends
- A way to stay healthy
- A way to stop cravings for sugar

Great, keep these benefits in mind *every time* you exercise or when you are starting to think about beginning to exercise.

That’s all for today.

Thanks for stopping by, and I look forward to seeing you again.

Following the demonstration, participants were given the survey, which took about 5-10 minutes to complete. Study staff then briefly reviewed the survey for possible unanswered items and placed it in a collection box. Participants received a US $25 gift card as remuneration for their time.

### Measures

The four-page survey included 28 items and did not collect any identifying personal information. Survey items assessed selected sociodemographic characteristics: zip code, age, biological sex, current grade level, and self-reported height and weight. We assessed cigarette smoking and depression history because of the inverse association shown with these variables and physical activity [24-26]. Our depression and lifetime smoking status questions were developed for this study but were similar to other validated single-item measures [27,28]. Participants were asked about their smoking history (ie, “Have you ever tried cigarette smoking, even one or two puffs?”) and depression history (ie, “Have you ever experienced depression now or in the past?”); each item had a *yes* or *no* response option. Exercise habits were assessed with the following question: “During the past seven days, on how many days were you physically active for at least 60 minutes per day? (Add up all the time you spent in any kind of physical activity that increased your heart rate and made you breathe hard some of the time),” to which they indicated 0-7 days [29].

Body mass index (BMI) was calculated for each participant using self-reported height and weight. Estimated household income was generated from the 2016 US American Community Survey by zip code (ie, postal code) [30,31]. Based on the sample distribution, household income was categorized into three categories: low, medium, or high.

The survey also included the eight-item, validated Technology Acceptance Scale (TAS). The TAS comes from the Davis Technology Acceptance Model [32-34]. Items assess (1) perceived usefulness and (2) perceived ease of use of new technologies. Each item was rated on a 5-point Likert scale ranging from 1 (*strongly disagree*) to 5 (*strongly agree*). Two items on the scale were reverse scored. Total possible scores ranged from 8 to 40, with higher scores indicating greater potential acceptance of the robotic trainer technology. For the current sample, Cronbach alpha was .70, suggesting relatively high internal consistency reliability.

Four additional questions assessed general reactions to robots, likelihood for using this technology for health promotion purposes, and reasons for engaging in physical activity. Participants were asked the following: “Do you think you and your friends are likely to use the robot to help you exercise?” and “Do you think there is a need for a robotic human trainer to help kids exercise?”; response options to each item were *yes* or *no*. In addition, adolescents were asked the following: “Where would you be most likely to use the robotic trainer?”; response options were *at school*, *local gym*, and *after-school program,* as well as *other,* in which participants could write in their response. Furthermore, adolescents were provided a list of four reasons why they might engage in physical activity and were asked to rank order each reason from most to least important (1-4): *to get or stay healthy*, *sports training*, *to have fun with friends*, and *to lose weight*.

### Statistical Analyses

SPSS Statistics for Windows (IBM Corp) was used to analyze the data; the data were summarized using univariate and bivariate statistics. Acceptance ratings—TAS individual items and total score—were summarized for the overall sample using descriptive statistics. The association of TAS total score with sociodemographic characteristics was examined using *t* tests for dichotomous variables of age group (12-14 versus 15-17 years), sex (male or female), racial minority status (yes or no), meeting physical activity national recommendations (yes or no), and depression history (yes or no). One-way analysis of variance (ANOVA) was used to examine the association of total TAS score with BMI, categorized as underweight, normal, or overweight, and estimated household income status, categorized as low, medium, or high. *P* values of .05 or less were used to denote statistical significance.

## Results

### Participant Sociodemographic Characteristics

Table 1 shows the sociodemographic characteristics of the 190 youth who participated in the study. Among them, 45.5% (86/189) were female (one person did not answer the question on sex), 56.8% (108/190) were white, and 36.8% (70/190) were African American or black. A total of 5.8% (11/190) of the sample reported Hispanic ethnicity. Approximately half of respondents (94/190, 49.5%) were between the ages of 12 and 14 years. Low-income household status was estimated for 28.4% (54/190) of the sample. Only about one-quarter (47/190, 24.7%) of participants met national recommendations for physical activity. The mean BMI was 21.8 kg/m^2^ (SD 4.0), 19.5% (37/190) of respondents were classified as overweight, and 18.4% (35/190) had experienced depression now or in the past. Very few reported they had ever tried cigarette smoking.

**Table 1:** Sociodemographic characteristics of adolescent survey participants (N=190).

Characteristic		Value
**Biological sex (N=189)^b^, n (%)**		
	Male	103 (54.5)
	Female	86 (45.5)
**Race, n (%)**		
	White	108 (56.8)
	Black or African American	70 (36.8)
	Asian	6 (3.2)
	American Indian or Alaska Native	3 (1.6)
	Native Hawaiian or other Pacific Islander	2 (1.1)
Ethnicity (Hispanic or Latino), n (%)		11 (5.8)
**Household income category^c^, n (%)**		
	Low (<US $58,056)	54 (28.4)
	Medium (US $58,056-US $70,145)	65 (34.2)
	High (>US $70,145)	71 (37.4)
**Age group, n (%)**		
	12-14 years	94 (49.5)
	15-17 years	96 (50.5)
**Grade levels, n (%)**		
	Middle school	73 (38.4)
	High school	117 (61.6)
Ever tried a cigarette (yes), n (%)		5 (2.6)
Experienced depression now or in the past (yes), n (%)		35 (18.4)
Meets national recommendations for physical activity (yes), n (%)		47 (24.7)
**Body mass index (kg/m^2^)**		
	Mean (SD)	21.8 (4.0)
	Range	14.4-37.6
	Percent overweight, n (%)	37 (19.5)

^a^Percentages are based on nonmissing data.

^b^One person did not answer the question on sex.

^c^Median household income in the United States and Rochester, MN, in 2016 was US $59,039 and US $65,195, respectively.

### Technology Acceptance Scale

Table 2 shows the mean and total TAS scores. The mean total score was a 32.8 (SD 4.2, range 12-40) out of a possible score of 40, indicating high technology acceptance. No statistically significant associations were found between TAS total score and participant sex, age group, racial minority status, participant-estimated neighborhood household income, meeting physical activity recommendations, BMI, or depression history (*P*>.05 for all).

We found that 67.8% (129/190) of participants agreed that they and their friends would be likely to use the robot to help them exercise; 77.8% (148/190) agreed that there is a need for a robotic human trainer to help kids exercise. When participants were asked where they think they would most likely use the robotic trainer, 71.1% (135/190) indicated a local gym such as the YMCA, 40.0% (76/190) reported in a school setting, and 46.8% (89/190) indicated at an after-school program; 71.1% (135/190) suggested *other* locations, all of whom wrote “at home.” When given a list of four reasons about why they engage in physical activity, 42.1% (80/190) of respondents reported that *to get or stay healthy* was the most important reason and 46.8% (89/190) indicated that *to lose weight* was the least important reason.

**Table 2:** Technology Acceptance Scale items and total scores^a^ (N=190).

Item	Score, mean (SD)^b^
1. The robot trainer was clear and easy to understand.	4.29 (0.83)
2. I would find it easy to ask the robot trainer something.	4.32 (0.85)
3. It would take a lot of effort to interact with the robot trainer.	3.52 (1.22)
4. I would feel confident interacting with the robot trainer.	4.22 (0.91)
5. I would find it easy to interact with the robot trainer.	4.17 (0.94)
6. The robot trainer could help to encourage me to exercise.	4.12 (0.94)
7. I would find it frustrating to interact with the robot trainer.	3.97 (1.18)
8. The robot trainer could be helpful for me when exercising.	4.19 (0.89)
Total score, mean (SD)	32.8 (7.8)
Total score, range	12-40

^a^All Items were rated on a 5-point Likert scale that ranged from 1 (*strongly disagree*) to 5 (*strongly agree*). Items 3 and 7 were reverse scored so that a higher score indicated less effort (item 3) or less frustration (item 7). The total score has a possible range between 8 and 40, with higher scores indicating greater acceptability of the robot technology.

^b^All eight items have an observed range of 1-5.

## Discussion

This preliminary study found that among a group of racially and socioeconomically diverse adolescents, potential receptivity to a human robotic-assisted trainer for delivering physical activity coaching was high, as evidenced by a mean score of 32.8 (SD 7.8) on the TAS. A previous study by our group found that the same robotic human-trainer technology was considered novel and acceptable as a potential tool for supervised exercise coaching among an adult population [22]. However, little is known about the feasibility and consumer uptake of such an approach among adolescents, a group that may benefit greatly from interventions aimed at increasing physical activity levels. Like other studies, only about a quarter of adolescents in our sample met national recommendations for physical activity [4]. We are encouraged by the finding that all sociodemographic groups represented in our sample endorsed similarly high potential receptivity to the robotic technology for ultimate considerations of intervention scalability and reach among minority adolescent populations, where the prevalence of obesity is highest.

This study has several strengths, including the conceptual framework, the use of a valid and reliable measure of technology acceptance, successful recruitment in multiple community settings serving minority adolescent populations, and a racially and socioeconomically diverse sample with equal representation from both boys and girls.

Limitations of this study include the use of a convenience sample. Some sample characteristics, such as low prevalence of cigarette smoking, may limit generalizability of the findings to other settings and populations. We did not specifically ask about the use of alternatives to cigarette smoking, including e-cigarettes, hookahs, or vaping, whose use may have been more prevalent [35]. To reduce participant burden, we used self-reported data about height and weight, but these data may be unreliable. Although reasonable for a pilot study, the sample size was insufficient for conducting multivariate analyses on the association of participant characteristics and TAS score; thus, findings are exploratory. Furthermore, we assessed acceptability using a brief, mock introductory coaching session and did not measure acceptability while delivering a specific exercise intervention. It is possible that the robotic trainer will not translate into an acceptable, effective, or useful mode of engaging adolescents in physical activity.

Many studies examined the effects of school-based interventions to promote exercise, but community-engaged interventions have yet to demonstrate effectiveness [36]. As schools place less emphasis on physical activity during the school day, delivering appropriate alternative exercise opportunities is important [37]. Given the increased connectivity with digital technologies among adolescents, such platforms are important to consider for delivering effective interventions that have already been shown to improve chronic disease parameters and adherence to physical activity regimens [38,39]. Evidence suggests that adolescents perform online searches about nutrition and fitness and download apps centered on these subjects more than any other group, indicating an interest in this topic among youth [40].

In contrast to digital health coaching through Web and mobile phone apps, robotic-assisted exercise coaching provides both dialogue support and primary task support in real time [41,42]. Moreover, the current prototype is different than Skype or videoconferencing intervention delivery formats because the robot device can move with and around the participant, providing instruction and correction of exercise form, reinforcement, and support, and the participant can remain hands-free [43].

This pilot study was the first step in determining likely acceptability of robotic-assisted exercise coaching among an adolescent population [9,10,44,45]. The high acceptability of the robotic-assisted trainer in our sample of adolescents suggests several next steps. Future research is needed to evaluate adolescent consumer preferences for robotic-assisted exercise coaching (eg, location, duration, supervised or structured, choice of exercise, and/or lifestyle activity focus), develop a social-cognitive-based intervention protocol, and evaluate feasibility and consumer uptake of the intervention among diverse youth.

With the prevalence of obesity among minority adolescents, combined with a lack of access to exercise facilities or appropriate guided exercise, robotic trainers may be one potentially valuable tool for helping to increase physical activity in these vulnerable populations [46-48]. Because the technology requires reliable Internet or wireless access, the robotic human trainer poses a unique challenge; however, when functioning properly, it has high scalability and a large potential to reach many people. In the future, it is worth exploring this approach to reach underserved populations in the context of conditions associated with poverty and health disparities, including diabetes and other chronic illnesses that may improve with behavioral modifications [47].

With the benefits of exercise well-documented for both mental and physical health, the growing obesity epidemic in youth, and youth preferences for technology, a human robotic trainer could prove a welcome and feasible strategy for promoting and delivering healthy exercise habits to adolescents.

## Abbreviations

ANOVA: one-way analysis of variance
BMI: body mass index
SMS: short message service
TAS: Technology Acceptance Scale
